# Fitz‐Hugh–Curtis syndrome: a diagnostic challenge

**DOI:** 10.1002/ccr3.1553

**Published:** 2018-06-02

**Authors:** Wei Jia, Fiqry Fadhlillah

**Affiliations:** ^1^ Education Department Northwick Park Hospital London UK; ^2^ Chelsea and Westminster Hospital London UK

**Keywords:** Emergency medicine, gastroenterology and hepatology, general surgery, obstetrics and gynecology

## Abstract

Diagnosis of Fitz‐Hugh–Curtis is challenging due to its rarity and its similar presentation to common intra‐abdominal conditions, such as cholecystitis or appendicitis. In our case, the adherent cecal and omental mass felt on examination were thought to be an appendiceal mass secondary to perforation, hence the patient underwent a diagnostic laparoscopy.

A 17‐year‐old woman presented with right‐sided upper and lower abdomen pain of several days duration, with no associated history of vaginal discharge or urinary symptoms. Examination findings revealed pyrexia and guarding in the right abdomen. Deep palpation revealed a mass in the right iliac region, thought to be an appendiceal mass.

Blood workup revealed raised inflammatory markers. Urine dipstick and pregnancy testing were negative.

The patient underwent a diagnostic laparoscopy. Operative findings revealed an adherent mass of cecum and omentum to the anterior abdominal wall (Fig. 1A), with an embedded appendix requiring dissection to fully visualize it (Fig. 1B). Examination of the pelvis revealed pus and grossly inflamed uterus, ovaries, and fallopian tubes consistent with pelvic inflammatory disease (Fig. 2A). Examination of the liver revealed adhesions of the liver capsules to the anterior abdominal wall (Fig. 2B), with a normal gallbladder. Based on these findings, a diagnosis of Fitz‐Hugh–Curtis syndrome was made 1. Microscopically, there was no inflammation within appendix. Pus swabs from the pelvis did not grow any organisms, possibly due to the antibiotics started before the swabs. Patient remained symptom free at 6‐week follow‐up.

**Figure 1:**
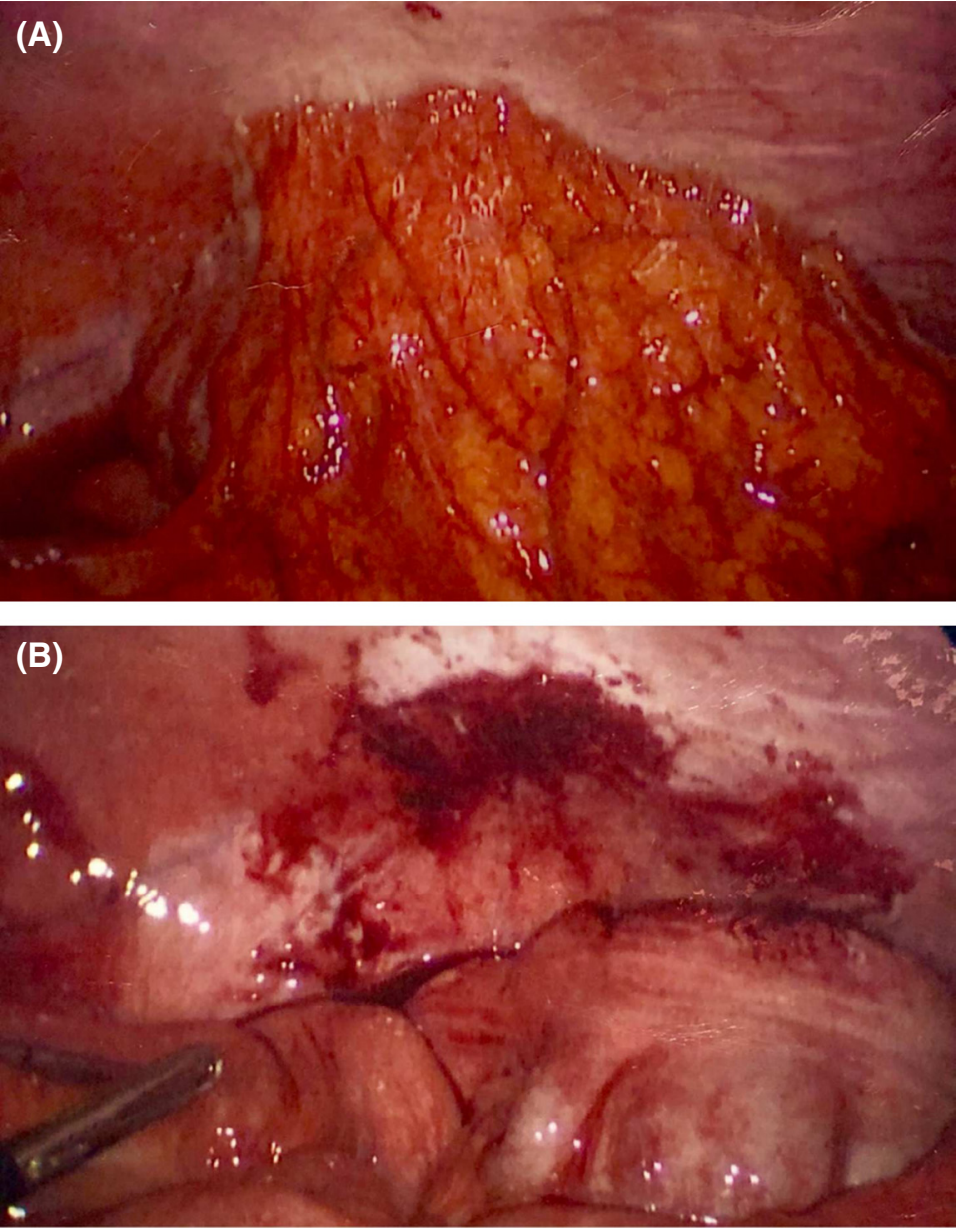
(A) Adherent mass of cecum and omentum to the anterior abdominal wall; (B) visualized cecum following dissection.

**Figure 2:**
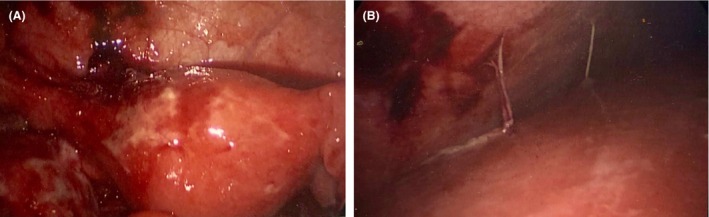
(A) Pus and grossly inflamed uterus, ovaries, and fallopian tubes consistent with pelvic inflammatory disease; (B) liver surface with violin string‐like adhesions to liver capsule 2.

## Authorship

WJ: was involved in obtaining consent and drafting of the manuscript. FF: was involved in writing of the manuscript. Both were involved in her clinical case.

## Conflict of Interests

None declared.
